# An Assessment of Thyroid Dysfunction and Related Parameters in Patients With Systemic Autoimmune Disorders

**DOI:** 10.7759/cureus.42783

**Published:** 2023-08-01

**Authors:** Akash Pawar, Prakash Joshi, Abhishek Singhai

**Affiliations:** 1 General Medicine, All India Institute of Medical Sciences Bhopal, Bhopal, IND; 2 Internal Medicine, Sri Aurobindo Medical College & PG Institute, Indore, IND

**Keywords:** prevalence, anemia, hypothyroidism, thyroid dysfunction, systemic autoimmune disorders

## Abstract

Background and objective

Systemic autoimmune disorders involve abnormal immune responses leading to tissue damage. Dysfunction of the thyroid gland due to autoimmune mechanisms is common in such disorders, which can cause either hypofunctioning or hyperfunctioning of the gland. This study aimed to investigate the prevalence of thyroid dysfunction among patients with various systemic autoimmune disorders.

Material and methods

This cross-sectional observational study included 110 adult patients either diagnosed with or having clinical/biological features of systemic autoimmune diseases. The patients underwent a detailed clinical history assessment, physical examination, and necessary investigations. Data were analyzed using IBM SPSS Statistics for Windows, Version 26.0. (IBM Corp., Armonk, NY).

Results

Among the 110 autoimmune disorder patients, 22.7% had thyroid dysfunction, specifically hypothyroidism, while 77.3% were euthyroid. Hypothyroidism was prevalent among patients with rheumatoid arthritis (RA, 20.3%), systemic sclerosis (SSc, 20%), ankylosing spondylitis (AS, 15.8%), and systemic lupus erythematosus (SLE, 54.5%). Moreover, 60% of patients were anemic, and the prevalence of anemia was higher among female patients and younger individuals.

Conclusions

This study showed a higher prevalence of thyroid dysfunction, particularly hypothyroidism, in patients with systemic autoimmune disorders. Female patients and younger individuals were more susceptible to autoimmune disorders, thyroid dysfunction, and anemia. These findings highlight the need for simultaneous screening and evaluation for thyroid dysfunction and anemia in systemic autoimmune disease patients, particularly in female patients and those of younger age groups.

## Introduction

Systemic autoimmune disorders involve abnormal immune responses to either self-antigens or other external antigens. These disorders emerge when the default mechanisms that regulate the tolerance of the immune system break down, resulting in self-reactivity that can damage tissue [1]. Examples of such disorders include rheumatoid arthritis (RA), systemic lupus erythematosus (SLE), systemic sclerosis (SSc), and ankylosing spondylitis (AS).

The thyroid gland, a crucial endocrine organ, can suffer dysfunction due to various causes, including autoimmune mechanisms. The three major thyroid dysfunctions are as follows: hypothyroidism, hyperthyroidism, and euthyroid sick syndrome. These autoimmune disorders can cause gland destruction, leading to thyroid hormone deficiency (hypothyroidism) or overproduction (hyperthyroidism) [2]. Such autoimmune dysfunctions are more prevalent among female patients and younger age groups compared to other demographics.

Several studies have reported the association between autoimmune disorders and thyroid dysfunction, which can lead to either hypofunctioning or hyperfunctioning of the thyroid gland [2]. The prevalence and type of this association vary among different autoimmune disorders [3], as well as among different gender and age groups. We conducted this study to investigate the prevalence of thyroid dysfunction among patients with various systemic autoimmune disorders.

## Materials and methods

This cross-sectional observational study involved 110 adult patients (more than 18 years of age at the time of the study) either diagnosed with or showing clinical/biological features suggestive of systemic autoimmune disease; it was conducted for a duration of 18 months during the period 2017-2018 at a tertiary care center in central India. Among 110 study patients, 77.2% were females and 22.8% were males. The study received approval from the institutional ethical committee (SAIMS/IEC/2016/17), and all participants provided informed written consent. The study classified patients into two age groups: patients aged 18-45 years (59.1%) and those older than 45 years (40.9%). Patients with a prior diagnosis of isolated thyroid disorders, those taking medications affecting their thyroid function, or those suffering from known chronic non-immune systemic disease were excluded. All patients underwent a detailed clinical history assessment, physical examination, and necessary investigations. Patients' symptoms had been correlated with clinical criteria and various biochemical abnormalities for diagnosis.

Clinical criteria used for the diagnosis of various autoimmune disorders were as follows: the American College of Rheumatology (ACR)/the European League Against Rheumatism (EULAR) 2010 Criteria for RA [4], EULAR/ACR Criteria for SLE [5], the Assessment of SpondyloArthritis International Society (ASAS) criteria for AS [6], and ACR/EULAR Criteria for SSc [7]. The standard normal ranges for thyroid function test parameters were 0.35-5.5 mIU/mL of thyroid-stimulating hormone (TSH), 0.8-2.0 ng/ml of triiodothyronine (T3), and 5.1-14.1 ug/dl of thyroxine (T4).

Data gathered from a customized proforma were entered into a Microsoft Excel sheet before being transferred to IBM SPSS Statistics for Windows, Version 26.0. (IBM Corp., Armonk, NY) for analysis. The study obtained correlation results among groups using the Karl Pearson correlation coefficient, with a p-value <0.05 deemed to be statistically significant.

## Results

Table 1 presents the distribution of autoimmune diseases among the 110 patients studied. Among these patients, 74 had RA, 11 had SLE, 19 had AS, five had SSc, and one had polymyositis. RA was the most prevalent autoimmune disease while polymyositis was the least prevalent.

**Table 1:** Distribution of patients with autoimmune diseases

Type of autoimmune disease	N (%)
Rheumatoid arthritis	74 (67.3%)
Systemic lupus erythematosus	11 (10%)
Ankylosing spondylitis	19 (17.3%)
Systemic sclerosis	5 (4.5%)
Polymyositis	1 (0.9%)
Total	110 (100%)

Of note, 85 (77.3%) of the patients were euthyroid, while 25 (22.7%) had thyroid dysfunction (specifically, hypothyroidism). This finding suggests that the prevalence of hypothyroidism in autoimmune patients is much higher than in the general population, which could be due to autoimmune thyroid gland involvement or a result of systemic autoimmune disease activity. Patients with hypothyroidism (25%) showed anti-thyroid peroxidase (anti-TPO) antibody positivity, indicating an autoimmune mechanism underlying the thyroid dysfunction.

Table 2 presents the gender distribution among autoimmune disease patients. Of the 110 patients, 77.3% were females and 22.7% were males. Hypothyroidism was observed in 24.7% of female patients and 16% of male patients.

**Table 2:** Autoimmune disease and thyroid dysfunction in patients by gender

Condition	Male, n (%)	Female, n (%)
Autoimmune disorder	25 (22.7%)	85 (77.23%)
Thyroid dysfunction	4 (16%)	21 (24.7%)

Table 3 presents a comparison of various parameters between euthyroid autoimmune disease patients (n=85) and hypothyroid autoimmune disease patients (n=25).

**Table 3:** Comparison of various parameters between euthyroid and hypothyroid autoimmune disease patients SD: standard deviation

Serial no.	Parameters	Study group: 110 patients		
		Group 1: euthyroid (n=85)	Group 2: hypothyroid (n=25)	P-value
		Mean ± SD	Mean ± SD	
1	Age, years	45.2 ± 13.1	45.6 ± 13.8	0.8948
2	Body mass index (BMI), kg/m^2^	21.7 ± 2.9	23.9 ± 2.7	0.0010
3	Weight, Kg	57.6 ± 7.6	62.8 ± 6.84	0.0046
4	Erythrocyte sedimentation rate (ESR), mm/hr	35.8 ± 21.1	36.2 ± 21.6	0.9341
5	Hemoglobin, g/dl	12.5 ± 1.9	9.6 ± 1.8	0.0001

When various parameters were evaluated and compared between 85 euthyroid patients and 25 patients with thyroid dysfunction (hypothyroidism), significant differences were found in BMI, weight, and hemoglobin (p-values: 0.0010, 0.0046, and 0.0001, respectively), but not in age and erythrocyte sedimentation rate (ESR) (p-values: 0.8948 and 0.9341, respectively). As for the age distribution of the 110 patients in the study, 59.1% were between the ages of 18 and 45 years, while 40.9% were over the age of 45 years. Patients in the younger age groups were more vulnerable to autoimmune disorders, except in the case of RA, where the majority of patients were more than 45 years of age.

**Figure 1:**
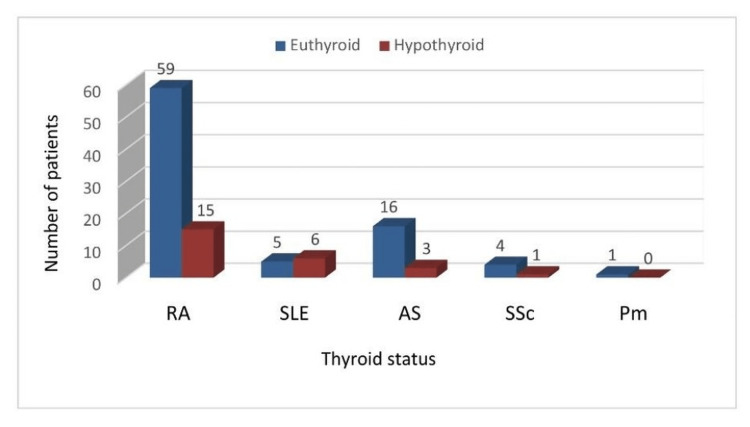
Distribution of patients according to thyroid dysfunction in different diseases RA: rheumatoid arthritis; SLE: systemic lupus erythematosus; AS: ankylosing spondylitis; SSc: systemic sclerosis; Pm: polymyosistis

Among RA patients (74), 15 (20.3%) were found to be hypothyroid. Among SSc patients (5), one (20%) was hypothyroid. Among AS patients (19), three (15.8%) were found to be hypothyroid. Among SLE patients (11), six (54.5%) were hypothyroid. The only patient with polymyositis was found to be euthyroid (Figure 1).

Anemia was identified in 66 (60%) of the 110 autoimmune patients in the study, while 44 patients (40%) did not have anemia. In our study, female patients had a higher prevalence of anemia than their male counterparts. Among anemic patients, 45 had normocytic normochromic anemia, 18 had microcytic hypochromic anemia, and three patients had megaloblastic anemia.

## Discussion

This study examined the prevalence of thyroid dysfunction in 110 patients with symptomatic systemic autoimmune disorders, spanning a period of 18 months. RA (67.3%) was found to be the most prevalent systemic autoimmune disorder among the cohort, which aligns with a prior study published in 2014 by Kumar et al. [8]. We identified 77.3% (n=85) of the patients as euthyroid, while 22.7% (n=25) of the patients were found to have thyroid dysfunction (hypothyroidism; no patient was found to have hyperthyroidism), for which thyroid hormone replacement was administered in oral tablet form (1.6 microgram/kg of body weight) along with autoimmune disorder-specific treatment. The prevalence of thyroid dysfunction in the general population is approximately 10% [9]. In this study, 20.3% of RA patients were hypothyroid, and 79.7% were euthyroid.

Except for SLE (54.5%), our study showed a prevalence of thyroid dysfunction ranging from 15% to 21% in various autoimmune disorders. These findings are consistent with previous studies conducted by Gokhale [9], Jaring et al. [10], Franco et al. [11], and Bliddal et al. [12]. Our study showed that autoimmune thyroid dysfunction is the most common endocrine dysfunction in systemic autoimmune diseases, with hypothyroidism being the most common and hyperthyroidism being the least common. Similar findings have been reported by Spoorenberg et al. [13], Antonelli et al. [14], and Lin et al. [15].

Our study found that 20.3% of RA patients had thyroid dysfunction, with hypothyroidism being the most common, which is similar to the findings of Elattar et al. (24%) [3], and Mosli et al. (22%) [16]. Thyroid dysfunction was found in 15% of AS patients, with hypothyroidism being the most frequent. Similar findings were reported by Tarhan et al. (13.8%) [17], Emmungil et al. (13%) [18], and Peixoto et al. (12.9%) [19]. We found a 54.5% prevalence of thyroid dysfunction in SLE, with hypothyroidism being the most common, which is similar to the findings in the study by Gokhale [9], though our findings differ from those of studies by Lin et al. [15] and Pan et al. [20]. The study by Antonelli et al. [14] observed a prevalence of thyroid dysfunction ranging from 14.6% to 21% in SSc patients, which is close to the 20% we found in our study. Further multi-center studies with larger sample sizes need to be conducted to more comprehensively assess the prevalence of thyroid dysfunction in SSc patients.

Our study found that females are more prone to autoimmune diseases and thyroid dysfunction than males: 85 (77.2%) of the patients in our study were female, with 21 (24.7%) of them being hypothyroid. In our study, 25 (22.8%) of the patients were male, with four (16%) being hypothyroid. Angum et al. [21] and Mulder [22] also found a similar female predominance. Rheumatological disorders primarily affect young and middle-aged populations. In our study, most patients (59.1%) were in the younger age group (18-45 years), consistent with the findings of May [23] and Vadasz et al. [24]. Among the 110 autoimmune disorder patients in our study, 60% were anemic, with the highest number of anemic patients found among those with RA and SLE. Moreover, female patients had a higher prevalence of anemia (63.5%) than male patients (48%).

Our study has a few limitations. Its observational and cross-sectional design prevented us from assessing the effects of clinical, biochemical, and drug-related factors on patient follow-ups to determine the impact of disease treatment on thyroid dysfunction and vice versa. The relatively small number of patients with some autoimmune diseases and the lack of established disease activity scores for some diseases also limited our ability to evaluate correlations between many parameters effectively.

## Conclusions

Our study found a higher prevalence of thyroid dysfunction, particularly hypothyroidism, in patients with systemic autoimmune disorders. We also observed that female patients and younger individuals are more susceptible to autoimmune disorders, thyroid dysfunction, and anemia. These findings stress the importance of simultaneous screening and evaluation for thyroid dysfunction and anemia in newly diagnosed or prediagnosed patients with systemic autoimmune diseases, particularly female patients and younger individuals. This approach advocates for early and prompt initiation of treatment for thyroid dysfunction, anemia, and the disease itself to slow the disease progression and manage thyroid dysfunction and anemia effectively.
